# Sonication versus peri-implant tissue culture in fracture-related infection: diagnostic performance and therapeutic consequences in a single-center cohort

**DOI:** 10.5194/jbji-11-555-2026

**Published:** 2026-08-26

**Authors:** Belal Abdo, Daniel Strack, Marcel Schäuffele, Tim Ludwig Tüngler, Can Imirzalioglu, Anita Windhorst, Moritz Fritzenwanker, Christian Heiss, Markus Rupp

**Affiliations:** 1 Department of Trauma, Hand and Reconstructive Surgery, Justus Liebig University Giessen, University Hospital of Giessen, Rudolf-Buchheim-Str. 7, 35392 Giessen, Germany; 2 Department of Cardiac and Vascular Surgery, Justus Liebig University Giessen, University Hospital of Giessen, Rudolf-Buchheim-Str. 7, 35392 Giessen, Germany; 3 Institute of Medical Microbiology, Justus Liebig University Giessen, University Hospital of Giessen, Schubertstr. 81, 35392 Giessen, Germany; 4 Experimental Trauma Surgery, Justus Liebig University Giessen, Aulweg 128, 35392 Giessen, Germany; 5 Institute of Medical Informatics, Justus-Liebig-University Giessen, Rudolf-Buchheim-Str. 6, 35392 Giessen, Germany; 6 Biruni University, Istanbul, Türkiye

## Abstract

**Introduction**: Fracture-related infections (FRIs) represent a severe complication and pose diagnostic challenges due to biofilm-associated microorganisms. While peri-implant tissue culture (PTC) remains the standard, its sensitivity is limited. Sonication of explanted osteosynthesis material has been proposed as a complementary diagnostic tool; however, its contribution remains controversial. The aim of this study was to compare sonication and peri-implant tissue culture and their impact on antimicrobial management.

**Methods**: In this retrospective single-center study, all patients undergoing implant removal for FRI between 2016 and 2023 were screened. Cases with peri-implant tissue cultures and sonication fluid cultures (SFCs) were included. The diagnostic yield and pathogen spectrum of PTC and SFC were compared, including analyses in patients receiving antibiotic therapy. The impact of microbiological findings on antibiotic management was evaluated.

**Results**: Of 287 screened cases, 157 patients met the inclusion criteria. Pathogen detection was achieved in 60.5 % using PTC and 57.3 % using SFC. Incorporating both methods increased the diagnostic yield to 70.1 %. Sonication identified additional pathogens in 17.8 % of cases and PTC in 22.9 %. Under antibiotic therapy, PTC demonstrated a slightly higher diagnostic yield than SFC. Antibiotic therapy was modified after revision surgery in 47.8 % of cases, while sonication alone influenced treatment decisions in 4.5 % of patients.

**Conclusions**: Neither PTC nor SFC alone achieved the diagnostic performance of their combined use. Combined testing improved diagnostic yield, supported targeted antimicrobial management, and reinforced the value of a multimodal microbiological approach to FRI.

## 1 Introduction

Fracture-related infections (FRIs) remain one of the most serious complications following surgical fracture care (Metsemakers et al., 2018; Rupp et al., 2024). According to the FRI Consensus Definition, FRI is confirmed by at least one confirmatory criterion, including a fistula, sinus tract, or wound breakdown communicating with the bone or implant; purulent drainage or pus encountered during surgery; phenotypically indistinguishable pathogens identified from at least two separate deep tissue or implant specimens; or histopathological confirmation according to the FRI Consensus criteria (Metsemakers et al., 2018). These infections increase morbidity, requiring surgical revision and prolonged hospitalization. Recent data from a German level I trauma center demonstrated a substantial increase in costs when FRI occurred, with cost increases ranging from 1.8- to 4.7-fold depending on fracture location (Walter et al., 2025).

A central pathophysiological characteristic is the formation of a biofilm on the surface of implanted osteosynthesis material (Metsemakers et al., 2018; Depypere et al., 2022). The diagnosis of biofilm-associated infection is a challenge. Conventional diagnostics are based on the cultivation of tissue samples obtained intraoperatively (Oliva et al., 2021; Silva et al., 2021). False-negative microbiological results occur in up to 35 % despite clinically manifest infection (Weinert-Stein et al., 2020). Furthermore, diagnostic sensitivity may be reduced by prior antibiotic treatment (Yano et al., 2014; Dudareva et al., 2018), whereas perioperative prophylactic antibiotics are not expected to significantly impair pathogen detection (Kapadia et al., 2016; Anagnostopoulos et al., 2018).

Sonication has become an established component of the diagnostic algorithm for periprosthetic joint infection (PJI). Multiple studies have consistently demonstrated that sonication fluid culture (SFC) provides higher sensitivity than conventional tissue cultures (Trampuz et al., 2007; Bellova et al., 2019; Watanabe et al., 2024).

Despite clinical application, the diagnostic accuracy of sonication in FRI and its implications for pathogen detection and antimicrobial management have not been fully clarified.

The present study therefore aimed to (i) compare the diagnostic accuracy of SFC with standard peri-implant tissue culture (PTC) in FRI, (ii) evaluate its diagnostic performance in patients receiving prior antibiotic therapy, and (iii) analyze therapeutic consequences.

## 2 Material and methods

**Figure 1:**
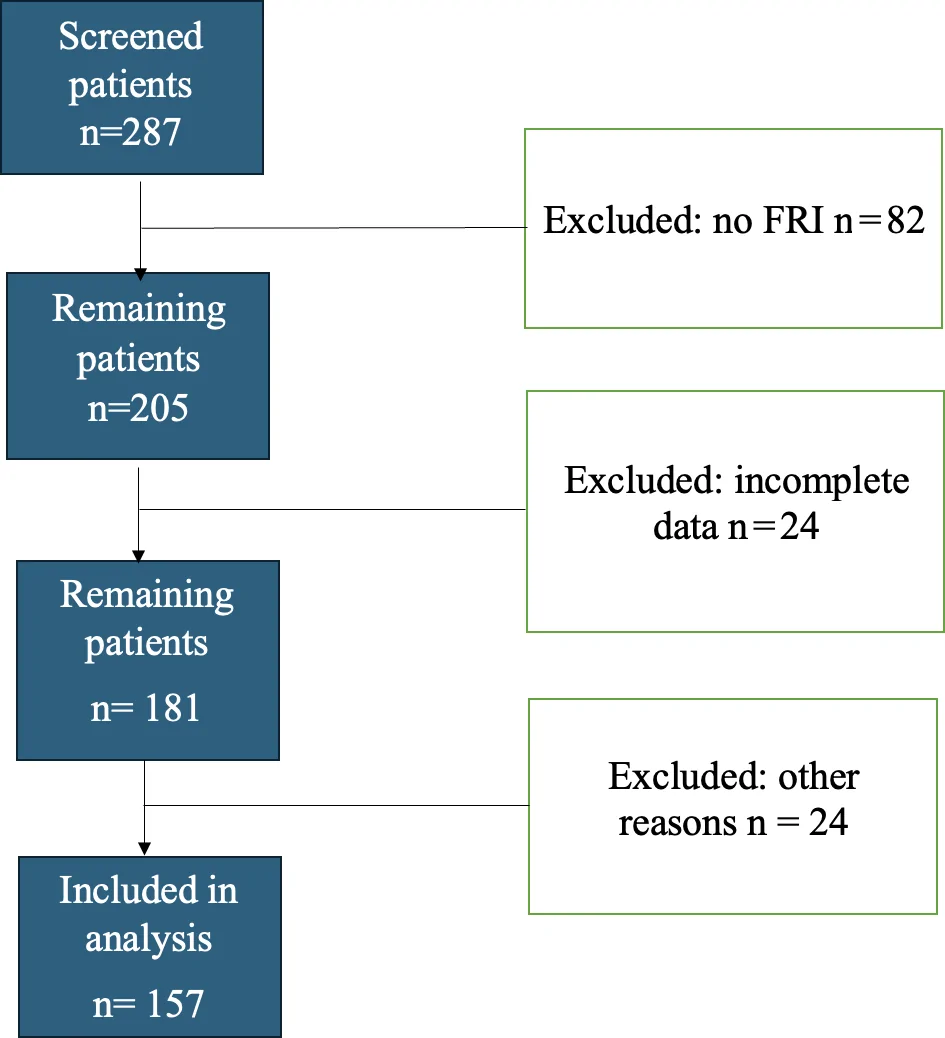
Flow chart of patient inclusion. Incomplete data refer to incomplete documentation or missing microbiological results. Other reasons refer to cases in which the analyzed material did not correspond to osteosynthesis components.

This retrospective, single-center observational study was conducted between 2016 and 2023 and is based on analysis of healthcare data extracted from the hospital's electronic patient data management system (PDMS). All data were pseudonymized in accordance with data protection regulations prior to analysis. The study was approved by the ethics committee (AZ 132/24) prior to analysis. During the study period, 287 cases were identified in which explanted material was analyzed using sonication. Patients were included if implant removal was required due to FRI criteria and if both PTC and sonication results were available. Cases were excluded if the analyzed material did not correspond to osteosynthesis components or if documentation was incomplete (Fig. 1).

For all included cases, clinical and microbiological data were collected retrospectively. These comprised patient age and sex, infection site, pathogen spectrum, monomicrobial or polymicrobial growth, multidrug resistance status, antibiotic treatment until 14 d before sample collection (not perioperative prophylaxis), number of prior surgical procedures at the affected site, and the type of removed implant material. In addition, it was documented whether a modification of the antibiotic regimen would have been possible and which diagnostic method prompted the therapeutic adjustment. To minimize potential bias caused by de-escalation of therapy or continuation of antibiotic treatment for reasons unrelated to the FRI, only treatment modifications specifically attributable to the FRI were considered. A change in therapy was defined as any escalation, de-escalation, or modification of the initial regimen and was attributed solely to sonication when the additional pathogen detected by this method could have been the direct cause of altering treatment. If pathogens detected by both methods showed identical susceptibility profiles, then the possible change was attributed to both.

PTCs were obtained using intraoperative tissue samples processed in the microbiology laboratory under standardized conditions. Tissue sampling was performed according to current guidelines. Sonication was performed according to protocol, with subsequent culture of the sonication fluid. The osteosynthesis material was placed into a container filled with 0.85 % NaCl and sonicated for 1 min (80 % intensity setting, BactoSonic BS 14, Bandelin, Germany). Of the resulting fluid, 50 mL was centrifuged (1500 g, 15 min, Megafuge 16, Thermo Scientific, USA). Supernatant was discarded, leaving 2–5 mL, resuspended by vortexing. From this solution, 200 µ L was inoculated into thioglycollate medium USP (Thermo Scientific) and tryptic soy broth (Merck Millipore, Germany). A total of 100 µ L was streaked onto Columbia sheep blood agar, chocolate agar (both ThermoFischer, Germany), and sabouraud gentamicin chloramphenicol agar (Biomerieux, France). These were incubated under aerobic conditions (36 °C, +5 % CO_2_). Another 100 µ L was streaked onto Schaedler anaerobe KV selective agar with lysed horse blood (Thermo Scientific) and incubated under anaerobic conditions. All media were incubated for 14 d or until growth was detected.

Descriptive statistics were used for data analysis. Categorical variables are presented as absolute and relative frequencies, while continuous variables are reported as absolute and relative frequencies, means, and medians, as appropriate. The final diagnosis of FRI according to the FRI Consensus Definition, established after revision surgery, served as the clinical reference standard. Diagnostic sensitivity was calculated as the proportion of confirmed FRI cases with pathogen detection by PTC, SFC, or their combination. Diagnostic yields of PTC and SFC were compared using McNemar's test for paired binary outcomes together with the proportion of agreement, as both methods were applied to the same patient. Sensitivity estimates are reported with two-sided 95 % confidence intervals calculated using the Wilson score method. Specificity was not assessed because only patients with confirmed FRI were included. All statistical analyses were performed using IBM SPSS Statistics, version 29.0.0.0 (IBM Corp., Armonk, NY, USA).

## 3 Results

### 3.1 Study population

Of the 287 cases in which sonication was performed at our institution, 157 patients were included in the final analysis. A total of 130 cases were excluded: 82 did not meet the criteria for fracture-related infection (FRI); 24 were excluded because of incomplete data, particularly missing information on the antibiotic regime; and 24 were excluded for other reasons (e.g., unclear labeling or missing tissue cultures) (Fig. 1). Missing data were mainly attributable to the transition to a new hospital patient data management system (PDMS).

The mean age of the included patients was 57.4 years. A total of 50 patients (31.5 %) were younger than 50 years, 33 (21.0 %) were between 50 and 59 years, and 74 (47.1 %) were 60 years or older. A total of 58 patients (36.9 %) were female, and 99 (63.1 %) were male (Table 1).

**Table 1:** Baseline characteristics.

	N=157	%
*Age*		
Mean/median/range	57.4/59/16–89	
< 50 years	50	31.5
50–59 years	33	21
≥ 60 years	74	47.1
*Sex*		
Female	58	36.9
Male	99	63.1
*Osteosynthesis materials*		
Plate osteosynthesis systems	96	61.1
Screws	29	18.5
Intramedullary nail	26	16.5
Anchor	1	0.7
K wire	1	0.7
Internal fixator systems	4	2.5
*Number of previous operations*		
Mean/median/range	1.6/1/1–11	
Antibiotic therapy^*^	51	32.5

^*^ Under ongoing antibiotic therapy or antibiotic treatment in the last 14 d (not perioperative prophylaxis).

Analysis of explanted implant types revealed a clear predominance of plate osteosynthesis systems (61.1 %) and screws (18.5 %).

The number of previous surgeries at the affected site varied. The mean number of prior operations was 1.6, and the range was 11 (Table 1).

Late infections ( > 10 weeks) were the most common presentation with 67 (42.7 %), followed by delayed infections (29.9 %) and early infections (27.4 %) (Table 2).

**Table 2:** Onset type of infection.

	Total		SFC +		SFC -		PTC +		PTC -	
	N=	%	N=	%	N=	%	N=	%	N=	%
Early < 2 weeks	43	27.4 %	23	25.6 %	20	29.9 %	25	26.3 %	18	29.0 %
Delayed 3–10 weeks	47	29.9 %	26	28.9 %	21	31.3 %	27	28.4 %	20	32.3 %
Late > 10 weeks	67	42.7 %	41	45.6 %	26	38.8 %	43	45.3 %	24	38.7 %

### 3.2 Sonication versus standard tissue culture

#### 3.2.1 Microbiological findings – SFC

Sonication yielded positive results in 90 of 157 cases (Table 4). Among these, 77 cases (85.6 %) were monomicrobial, while 13 (14.4 %) were polymicrobial. MRSA was detected in four cases (4.4 %), and one case (1.1 %) involved an MDR *Enterobacterales* strain (Table 5).

In total, 105 microbial isolates were detected across the 90 positive cases. The pathogen detection by this method was 57.3 % (Table 4).

#### 3.2.2 Microbiological findings – PTC

Conventional intraoperative microbiological diagnostics yielded pathogens in 95 cases (60.5 %) (Table 4). Among the positive cultures, 75 (78.9 %) were monomicrobial and 20 (21.1 %) polymicrobial. MRSA was identified in four cases (4.2 %), and one case (1.1 %) involved an MDR *Enterobacterales* strain (Table 5).

**Figure 2:**
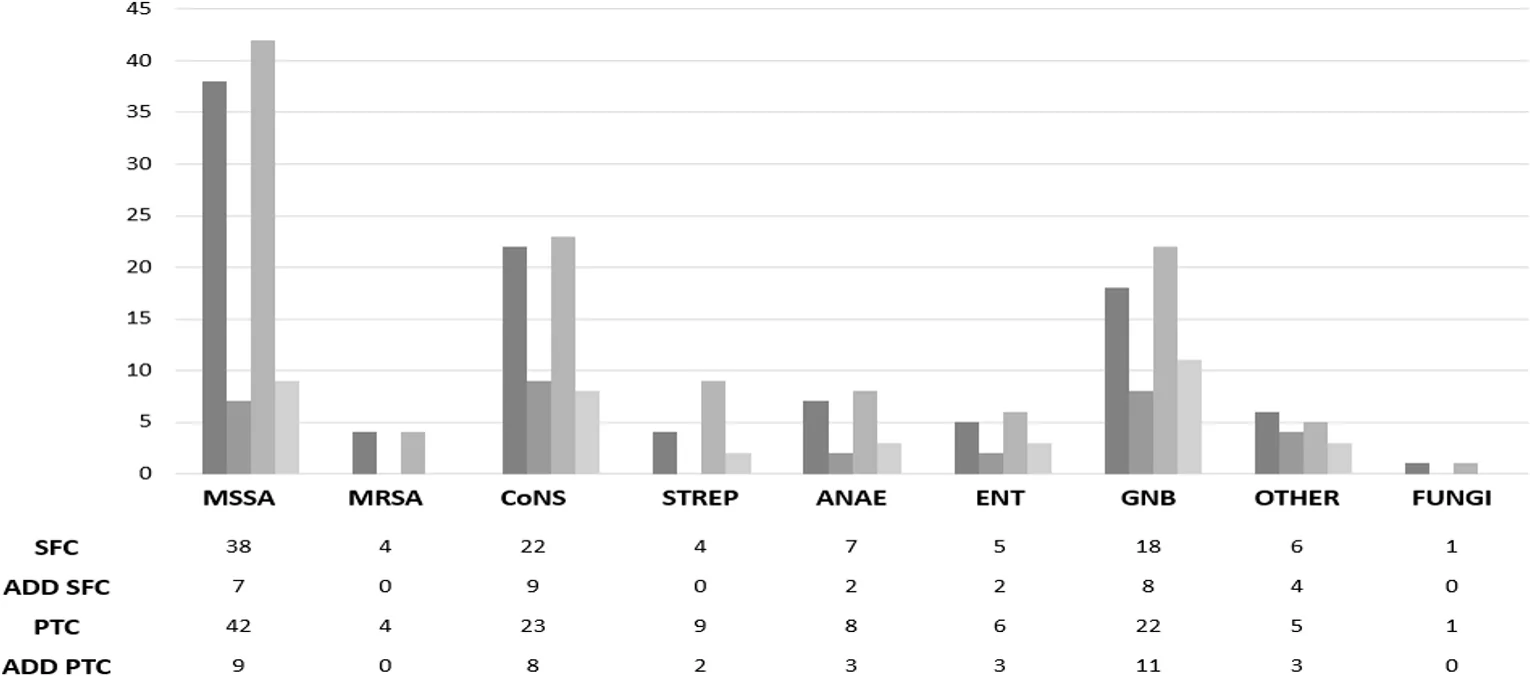
Distribution of pathogens: methicillin-sensitive staphylococcus aureus (MSSA), methicillin-resistant staphylococcus aureus (MRSA), coagulase-negative staphylococcus (CoNS), streptococcus (strep), anaerobic bacteria including cutibacterium, finegoldia magna (anae), enterococcus (ENT), Gram-negative bacilli (GNB), other (incl. *Corynebacterium* spp.) sonicate fluid culture (SFC), additional pathogen in sonicate fluid culture (ADD SFC), peri-implant tissue culture (PTC), and additional pathogen in peri-implant tissue culture (ADD PTC).

A total of 120 microbial isolates were detected across the 95 positive cases (Fig. 2).

### 3.3 Additional diagnostic value of sonication and impact of ongoing antibiotic therapy

At the time of implant removal, 51 of the 157 patients were under ongoing antibiotic therapy or had been under antibiotic treatment in the last 14 d (Table 1). Perioperative prophylaxis was not classified as therapy.

In 28 cases (17.8 %), SFC identified additional pathogens that were not detected by PTC. Of these, 9 were under antibiotic therapy (32.1 %). In 15 cases (16.7 %) SFC alone identified a pathogen (Table 4).

Conversely, in 36 cases (22.9 %), additional pathogens were detected exclusively by PTC. Of those, 17 were under antibiotic therapy (47.2 %). In 20 cases (21.1 %) PTC alone identified a pathogen (Table 4).

A total of 12 of the 47 double-negative cases (25.5 %) were receiving antibiotic therapy at the time of sampling or in the 14 d before the operation (Table 4).

No significant differences in pathogen detection were observed between PTC and SFC. Likewise, subgroup analyses stratified by time since surgery and ongoing antibiotic therapy revealed no significant differences between the two diagnostic methods (Table 3).

**Table 3:** p values.

	p values^a^	Portion of agreement
PTC vs. SFC^a^	p=0.499	0.78
Early < 2 weeks	p=0.774	0.72
Delayed 3–10 weeks	p=1.0	0.85
Late > 10 weeks	p=0.804	0.76
*Antibiotic therapy* ^b^		
PTC vs. SFC	p=0.238	0.65
Early < 2 weeks	p=0.453	0.63
Delayed 3–10 weeks	p=0.375	0.69
Late > 10 weeks	p=1.0	0.63

^a^ McNemar's test for paired comparison, ^b^ under ongoing antibiotic therapy or antibiotic treatment in the last 14 d (not perioperative prophylaxis).

In total, 110 cases (70.1 %) showed a positive finding detected by either method. The overall pathogen detection by both methods combined was 70.1 % (Table 4).

**Table 4:** PTC vs. SFC vs. PTC + SFC.

	PTC +		PTC -		SFC +		SFC -		PTC / SFC +		PTC / SFC -	
	N=	%	N=	%	N=	%	N=	%	N=	%	N=	%
Sensitivity	95	60.5	62	39.5	90	57.3	67	42.7	110	70.1	47	29.9
(95 % CI)^a^		(52.4–68.2)				(49.2–65.2)				(62.3–77.1)		
Antibiotic therapy^b^	33	34.7	18	29.0	27	30.0	24	35.8	39	35.5	12	25.5
Sensitivity (95 % CI)^a^		64.7 (50.0–77.6)				52.9 (38.5–67.1)				76.5 (62.5–87.2)		
Add pathogen	36	22.9			28	17.8						
Antibiotic therapy^b^	17	47.2			9	32.1						
PTC -					15	16.7 %						
SFC -	20	21.1 %										

^a^ CI (95 % confidence interval); ^b^ under ongoing antibiotic therapy or antibiotic treatment in the last 14 d (not perioperative prophylaxis).

**Table 5:** Drug resistance and polymicrobial growth.

	PTC		SFC		PTC + SFC	
	N=	%	N=	%	N=	%
Mono growth	75	78.9	77	85.6	89	80.9
Polymicrobial growth	20	21.1	13	14.4	21	19.1
No MDRO^a^	90	94.7	85	94.4		
MDR-E^b^	1	1.1	1	1.1	1	0.9
MRSA^c^	4	4.2	4	4.4	4	3.6

^a^ Multidrug-resistant organism (MDRO), ^b^ Multidrug-resistant enterobacterale (MDR-E), ^c^ Methicillin-resistant staphylococcus aureus (MRSA).

### 3.4 Clinical consequences

Following microbiological results, antibiotic therapy was modified in 75 cases. Of these, 61 modifications (38.9 %) were based on concordant findings from both conventional diagnostics and sonication, while in 7 cases (4.5 %), adjustments were driven exclusively by sonication results and in another 7 cases (4.5 %) solely by conventional culture findings. In 82 cases (52.2 %), no change in antibiotic therapy was made.

A potential modification of antibiotic therapy would have been possible in 93 cases. Of these, 68 cases (43.3 %) were based on concordant results of both diagnostic methods, while 13 cases (8.3 %) and 12 cases (7.6 %) were attributable exclusively to sonication and conventional culture findings, respectively. In 64 cases (40.8 %), no therapeutic adjustment would have been feasible (Table 6).

**Table 6:** Changes and possible changes in antibiotic regimen.

	Change		Possible change	
	N=	%	N=	%
PTC	7	4.5	12	7.6
SFC	7	4.5	13	8.3
SFC + PTC	61	38.9	68	43.3
No change	82	52.2	64	40.8

## 4 Discussion

In this study, PTC and SFC showed similar yields (60.5 % vs. 57.3 %), while their combined use increased pathogen detection to 70.1 %. Each method identified pathogens missed by the other. Most removed implants were plate osteosynthesis systems, and most patients had undergone only the initial fixation.

### 4.1 Diagnostic accuracy of SFC compared with PTC in FRI

SFC detected additional pathogens in 17.8 % of cases, whereas 22.9 % of isolates were identified exclusively by PTC, indicating that their combination provides the highest diagnostic yield. Onsea et al. (2018) found that SFC may serve as a useful adjunct to PTC without strong evidence for its superiority. Although most studies reported higher diagnostic sensitivity for sonication, some favored PTC over SFC. Reported sensitivities for SFC vary widely, ranging from approximately 52 % to 100 %, depending on the study population, infection type, and microbiological technique used. In contrast, reported sensitivities for conventional PTC generally range from approximately 56 % to 72 %. These data highlight variability in diagnostic performance and emphasize that neither method consistently demonstrates superior sensitivity. When both methods are combined, diagnostic sensitivity may increase substantially, reaching up to 97 % (Holinka et al., 2011; Yano et al., 2014; Portillo et al., 2015; Dudareva et al., 2018; Trenkwalder et al., 2023; Otchwemah et al., 2025). Trenkwalder et al. (2023) reported that combined SFC and PTC improved diagnostic sensitivity in septic and aseptic nonunion. In their analysis, SFC from membrane filtration (sensitivity 52 %, specificity 93 %) showed lower sensitivity than tissue culture (69 %, 96 %) but higher than that of histopathology (14 %, 87 %), supporting our findings.

Dudareva et al. (2018) reported a higher sensitivity for tissue culture (64 %) than sonication (45 %) in orthopedic-device-related infections, while the combined use of both methods improved the sensitivity to 68 %. Sonication was considered complementary rather than superior and showed similar sensitivity mainly in infections with less virulent pathogens. This is consistent with the findings of Puig-Verdié et al. (2013) that sonication may be more relevant in delayed infections, whereas tissue cultures appear more relevant in early infections.

SFC and PTC showed overlapping pathogen spectra, with MSSA and CoNS predominating in both methods, consistent with Bellova et al. (2021). While Trenkwalder et al. (2023) reported CoNS and *Cutibacterium acnes* to be most common, Unsworth et al. (2024) found *Staphylococcus aureus*, Gram-negative bacilli, and CoNS to be predominant pathogens.

Numerically in our study, polymicrobial infections were more frequently detected by PTC (21.1 % vs. 14.4 %), although this difference was not statistically significant, whereas Gram-negative pathogens, anaerobes, and fungi were detected with similar frequency. Unsworth et al. (2024) reported polymicrobial infections in 32 % of FRIs.

Our findings indicate that the combined use of SFC and PTC increased pathogen detection; however, nearly 30 % of clinically confirmed FRIs remained culture negative. This emphasizes that microbiological methods should be interpreted within the framework of the established FRI diagnostic criteria, which integrate clinical, intraoperative, histopathological, and microbiological findings.

### 4.2 Additional diagnostic value of sonication and impact of ongoing antibiotic therapy

The influence of prior antibiotic therapy on the diagnostic performance of microbiological methods remains controversial. In our cohort, ongoing antibiotic therapy was not associated with a statistically significant difference in pathogen detection between PTC and SFC. Although PTC showed a numerically higher diagnostic yield in patients receiving antibiotics, this finding should be interpreted with caution given the limited sample size and the absence of statistical significance.

Previous studies have reported heterogeneous results. Dudareva et al. (2018) observed higher sensitivity for tissue cultures than sonication in patients without prior antibiotic therapy (71 % vs. 57 %). In patients receiving antibiotics within 14 d before surgery, sensitivities decreased to 58 % for tissue culture and 48 % for sonication. Yano et al. (2014) similarly demonstrated an overall reduction in microbiological sensitivity following antibiotic exposure, with tissue cultures appearing more affected than sonication. In contrast, Velasquez et al. (2024) found no significant influence of prior antibiotic therapy on sonication fluid culture yield.

Furthermore, the landmark study by Trampuz et al. (2007) demonstrated that sonication improved pathogen detection in periprosthetic joint infection, particularly after previous antibiotic treatment, which has been attributed to the persistence of biofilm-associated bacteria on implant surfaces.

Taken together, the available evidence suggests that the impact of antibiotic therapy on microbiological diagnostics differs between studies and may depend on factors such as the underlying infection entity, pathogen spectrum, biofilm maturity, duration and type of antibiotic treatment, and methodological differences in sampling and culture techniques. As the present study exclusively included patients with FRI, direct comparison with studies on PJI should be made with caution. Nevertheless, our findings support the complementary use of PTC and SFC, as combining both methods increased overall pathogen detection irrespective of prior antibiotic therapy.

### 4.3 Clinical consequences

No clear difference between SFC and PTC was observed regarding antibiotic modification, as each method alone led to therapy changes in 4.5 % of cases. Most adjustments were based on combined microbiological findings. Sonication had a direct clinical impact by identifying pathogens missed by conventional diagnostics and guiding antibiotic modification. Corrigan et al. (2022) concluded that neither the time from injury nor presumed microbiological patterns should guide FRI treatment, particularly given the occurrence of polymicrobial infections at all time points. Empiric therapy for culture-negative cases should follow the spectrum of pathogens observed in culture-positive infections. In our study the antibiotic regime was unchanged in 52.2 % of patients.

Microbiological results need to be integrated and should not be interpreted alone. Within the current FRI consensus framework, microbiological evidence supports diagnosis and guides antimicrobial management (Metsemakers et al., 2018; Govaert et al., 2020).

## 5 Limitations

The following limitations should be considered. First, a substantial proportion of potentially eligible cases (130 of 287, 45.3 %) was excluded due to incomplete documentation. As the missingness pattern was not formally analyzed, a systematic selection bias cannot be excluded. Second, due to the retrospective design, the exact number of intraoperative tissue samples could not be verified because multiple specimens were often submitted under the same laboratory request. Although tissue sampling was performed according to institutional practice and current guidelines, variability in the number of tissue samples may have influenced the diagnostic performance of PTC. Third, the duration of infection prior to sampling was not analyzed, although pathogen virulence may influence microbiological spectra (Puig-Verdié et al., 2013; Tani et al., 2018). Fourth, heterogeneity in sample types and the absence of a gold standard limited comparability. Furthermore, because microbiological culture constitutes part of the FRI Consensus Definition, incorporation bias cannot be completely excluded when interpreting the reported sensitivity estimates. Therefore, diagnostic yield may represent a more appropriate outcome measure for assessing the additional value of the microbiological methods. No formal sample size calculation was performed prior to data collection. Additionally, the lack of a predefined protocol for interpreting microbiological results and guiding therapeutic decisions reflects real-world clinical practice but may have contributed to variability. Fifth, sonication of large implants may be prone to contamination. Finally, the single-center and retrospective design limits generalizability.

## 6 Conclusion

In conclusion, neither peri-implant tissue culture nor sonication fluid culture alone achieved optimal pathogen detection in FRI. Sonication complements tissue culture rather than replacing it. The combined use of both methods improves pathogen detection, highlighting the potential value of a multimodal diagnostic approach.

## Data Availability

The datasets generated and/or analyzed during the current study are not publicly available due to data protection regulations but are available from the corresponding author upon reasonable request.

## Appendix A Abbreviations

**Table:**

AO	Arbeitsgemeinschaft für Osteosynthesefragen
CoNS	Coagulase-negative staphylococci
FRI	Fracture-related infection
GNB	Gram-negative bacilli
MDR-E	Multidrug-resistant *Enterobacterales*
MDRO	Multidrug-resistant organism
MRSA	Methicillin-resistant *Staphylococcus aureus*
MSSA	Methicillin-sensitive *Staphylococcus aureus*
PDMS	Patient data management system
PTC	Peri-implant tissue culture
SFC	Sonicate fluid culture
